# What do we know: positive impact of hip-hop pedagogy on student's learning effects

**DOI:** 10.3389/fspor.2024.1490432

**Published:** 2025-01-06

**Authors:** Xi Ling, Yuanyuan Chen, Shixin Zhao, Dongping Zheng

**Affiliations:** ^1^ Faculty of Education, Silpakorn University, Sanam Chandra Palace Campus, Nakhon Pathom, Thailand; ^2^ Physical Education Department, Fuzhou Institute of Technology, Fuzhou, Fujian, China; ^3^ International Education Center, Thailand National Sports University, Bangkok, Thailand; ^4^ School of Physical Education, Fujian Polytechnic Normal University, Fuzhou, Fujian, China

**Keywords:** positive impact, hip-hop pedagogy, hip-hop culture, student, learning effect

## Abstract

Over the last decade, hip-hop pedagogy has received a lot of attention in the field of education because of its significance in improving students’ learning effects. This review articulates the current understanding of the positive impact of hip-hop pedagogy on students’ learning effects within publications, an under-researched area. Based on a review of previous studies, this review innovatively examines six major elements of hip-hop pedagogy (DJ, MC, cypher, breakdance, knowledge, and graffiti) and concludes that the six major elements of hip-hop pedagogy can, in terms of the four dimensions of learning motivation, engagement, learning and memorization, and critical thinking, improve students’ learning effects. Hip-hop pedagogy is an all-encompassing pedagogy that is popular with youth and deserves more in-depth research by hip-hop educators in the future.

## Introduction

“Those who merely know about something are not as proficient as those who take an interest in it, and those who take an interest in it are surpassed by those who find joy in it.” (Confucius, The Analects)

In recent years, hip hop as a form of active living, is being incorporated into student learning, and the use of hip hop pedagogy to improve students’ learning effect is receiving increasing attention (1). Numerous studies have demonstrated that hip-hop pedagogy can enhance students’ learning experiences (2), empower students with greater agency in the learning process (3) and foster critical thinking (4, 5). Hip-hop educators Zhi Yang and his colleagues proposed three flexible definition templates for hip-hop pedagogy that are richer than other studies (6), which is that

Hip-hop pedagogy is defined as learning activities through hip-hop elements in formal or informal learning environments. The dynamic feature of cipher allows students to choose whether to learn in a formal or informal learning space at their own pace (6, p. 05).

While the literature has discussed the use of hip-hop pedagogy in the classroom, fewer studies have focused on the positive impact on students’ learning effect (2, 7, 8). Existing literature has been lacking in documenting which hip-hop elements of hip-hop pedagogy have a positive impact on improving students’ learning effect, and the impact of different hip-hop elements on students’ learning effect (9). Second, there is a lack of research on the positive impact of non-hip-hop elements in the hip-hop pedagogy on students’ learning effect (10). For example, the non-hip-hop element, cypher, has been documented only one-sidedly for its role in communication and sharing. Existing literature describes cypher as a measure of hip-hop pedagogy that guides student learning, ignoring the benefits of cypher in improving students’ learning effect.

Although the literature separately mentions the role of hip-hop pedagogy in interpersonal communication and intercultural communication (11), learning motivation (12), interest (13–15), sense of belonging (16), engagement (17), learning and memory (18, 19), and positive effects in terms of critical thinking (20–22). However, these studies typically focus on a single aspect of hip-hop pedagogy, such as emphasizing only the role of hip-hop music or rap, and lack a comprehensive analysis of the key factors from a framed perspective of the entire hip-hop element.

A few studies have explored the role of hip-hop pedagogy as an educational tool in improving students’ learning effect, but only on the positive effects of a single aspect, such as engagement or critical thinking (2, 7, 8). Although Laforgue-Bullido et al. (23) explored the application of hip-hop pedagogy in educational practice, but there is no in-depth discussion of what improves students’ learning effect. In addition, the time interval of Laforgue-Bullido et al.'s literature collection was limited to relevant literature published during 2012-2022, which may result in missing literature searches. Notably, although the study by Laforgue-Bullido et al. (23) did confirm that hip-hop pedagogy the potential benefits of hip-hop culture as an educational tool, but the performance on students’ learning effect was also limited to social awareness, action capabilities, critical thinking, social skills and psychosocial factors. Laforgue-Bullido et al.'s were not sufficient to draw firm conclusions about hip-hop pedagogy improving students’ learning effect. Because their study only used “hip-hop” or “rap” AND “pedagogy” or “education” when searching for keywords (23, p. 05), in fact, there may have been more than these search terms, and the search results may have affected the completeness of the conclusions.

In short, existing research does not reveal how hip-hop pedagogy enhances student learning, nor does it provide an integrative description of the positive effects that hip-hop pedagogy produces. The lack of clear evidence to support this may makes it difficult for teachers to identify and build on the elements of the hip-hop pedagogy that are effective, and may make it difficult to persuade school management and parents to accept this innovative approach to teaching and learning. Students may question the effectiveness of the hip-hop pedagogy, affecting their acceptance and understanding of classroom content.

In this context, we want to activate a lively discussion about positive impact of hip-hop pedagogy on student's learning effect, a reintegration of the elements of hip-hop pedagogy and a hip-hop pedagogy on student's learning effect. We argue that the elements of hip-hop pedagogy should not only contain the five hip-hop elements in the traditional sense (19, 24, 25), but should also include cypher (6).

In this review, we first analyze the pedagogical components of hip-hop pedagogy that improve student's learning effect in order to more visually represent these key factors, and then describe the manifestations of the positive impact of hip-hop pedagogy on student's learning effect. Specifically, it addresses the following key questions:

Research question 1(RQ1): What elements of hip-hop pedagogy are appropriate for improving students’ learning effect?

Research question 2(RQ2): What are the effects of hip-hop pedagogy on students’ learning effect?

By answering these two questions, this review aims to explore the key elements and positive impacts of hip-hop pedagogy in improving students’ learning effect, and is an important reference for students, teachers, parents, and school administration. It demonstrates how hip-hop pedagogy improving students’ learning effect through hip-hop elements that enhance student learning motivation, engagement, learning and memorization, and critical thinking. Teachers can use this research to design more culturally relevant and interactive lessons, parents support their children's learning by understanding the benefits of hip-hop pedagogy, school administrations provide a basis for promoting the implementation of hip-hop pedagogy, and researchers can apply research findings to teaching practices and design more culturally relevant and interactive curricula.

## Method

It is aimed to construct a review on positive impact of hip-hop pedagogy on students’ learning effect. Search terms for hip-hop pedagogy, learning effect and students. This review was based on published articles and followed the guidelines of PRISMA for research procedures (26).

### Search strategy

Previous studies on impact of hip-hop pedagogy on students’ learning effect were searched as follows. The studies published in international journals were searched in electronic databases of Web of Science and Scopus, the search term “hip-hop pedagogy” is derived from the study of Wei et al. (6), and the term “learning effect” is derived from the study of Tan et al. (27). Search methodology terms and fields used to locate articles is detailed in Table 1.

**Table 1:** Search methodology terms and fields used to locate articles.

Search methodology keywords and fields used to locate articles		
Web of science	Search Terms 1	TI = (“Learning Outcome” OR “Learning Effectiveness” OR “Learning Achievement” OR “Learning Effect” OR “Learning Performance” OR “Learning Gain” OR “learning outcome” OR “learning achievement” OR “learning performance” OR “academic achievement” OR “academic performance” OR “learning perception” OR “learning attitude” OR “learning motivation” OR “learning experiences” OR “satisfaction” OR “perception”)
	Search Terms 2	ALL = (“hip hop pedagogy” OR “hip-hop pedagogy” OR “hip-hop based education” OR “hip-hop element” OR “hip-hop culture” OR “hiphop education” OR “hip-hop education” OR “Hiphop education” OR “Hip-Hop education” OR “pedagogy of education” OR “pedagogy” OR “pedagogical orientation” OR “pedagogical practices” OR “Hip-Hop Based Education”)
	Search Terms 3	ALL = (“student” OR “learner”)
Scopus	Search Terms 1	TITLE-ABS-KEY (“Learning Outcome” OR “Learning Effectiveness” OR “Learning Achievement” OR “Learning Effect” OR “Learning Performance” OR “Learning Gain” OR “learning outcome” OR “learning achievement” OR “learning performance” OR “academic achievement” OR “academic performance” OR “learning perception” OR “learning attitude” OR “learning motivation” OR “learning experiences” OR “satisfaction” OR “perception”))
	Search Terms 2	(TITLE-ABS-KEY (“hip hop pedagogy” OR “hip-hop pedagogy” OR “hip-hop based education” OR “hip-hop element” OR “hip-hop culture” OR “hiphop education” OR “hip-hop education” OR “Hiphop education” OR “Hip-Hop education” OR “Hip-Hop Based Education” OR “hiphop pedagogy” OR “hiphop” OR “hip-hop” OR “hip hop”))
	Search Terms 3	(TITLE-ABS-KEY (“student” OR “learner”))

### Inclusion and exclusion criteria

Table 2 lists the terminology of the literature review and reasons for inclusion/exclusion. The inclusion criteria see Table 2.

**Table 2:** Inclusion and exclusion criteria.

Inclusion criteria	Exclusion criteria
Investigation of at least one aspect of improving learning effect, such as Cultural identity, critical thinking, and engagement.	Book, book chapters, and conference papers
Hip-hop pedagogy as intervention measure, such as DJ, breakdance, MC, knowledge, cypher, and graffiti.	The sample does not involve student groups
English language	No hip-hop pedagogy content or learning efficiencies involved
Journal articles published	Systematic literature review

**Figure 1:**
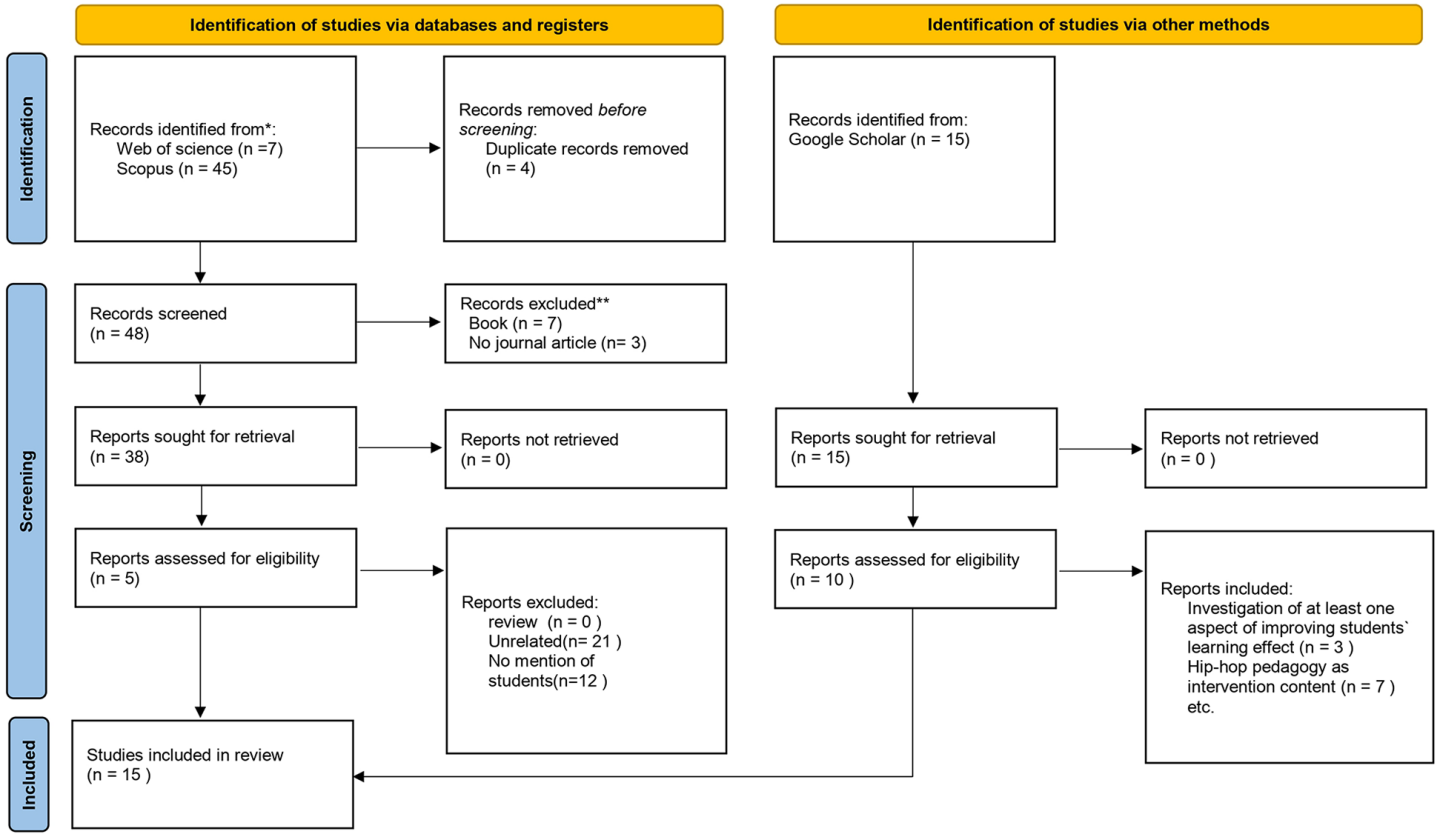
Literature review and incorporation flowchart.

Figure 1 showing the literature review and incorporation diagram (26). The diagram has been modified to show what is involved in the various stages of the literature review process to identify and screen the peer-reviewed research literature.

## Result

This review used thematic analysis for data analysis. After the initial review, 52 articles were selected. In the next step, 52 articles were reviewed in full text to determine if the literature met the inclusion criteria for this study. In this review, 47 articles that did not meet the criteria, such as no mention students, unrelated, books, and duplicate, were excluded from the study. Additionally, we used Google scholar as a triangulation to determine if articles in related fields still existed, 15 additional articles were added, and after screening for inclusion criteria, 10 articles were selected. Ultimately, 15 articles were included in the data analysis.

### Data extraction

We created a data extraction table by extracting the following data from the included articles. The content included: Full reference, country, research question or focus, sample and research duration, methods and data collection, and RQ1 and RQ2 (See Table 3 for details).

**Table 3:** Detailed summary of results.

No	Full reference	Country	Research question or focus	Sample and research duration	Methods and data collection	RQ1 and RQ2
1	(28)	USA	How hip-hop culture impacts the undergraduate learning experience.	*n* = 11. College. 6 females and 5 males. Age from 17–23 Duration is not clearly stated.	1. Qualitative phenomenological approach. 2. Semi-structured individual interviews to collect data.	**RQ1:** Concerns about hip-hop Culture Influencing the Educational Experience of Undergraduate Students. **RQ2:** Hip-hop pedagogy had a positive impact on the students’ learning experiences, particularly in the areas of socialization, personal expression, goal-setting, and cultural appreciation.
2	(29)	USA	To explore the experiences and perspectives of the majority of students of color in a hip-hop course led by a white music teacher.	*n* = 18. High school. 15–19 years 18 weeks.	1. An exploratory study that is based on qualitative research methods and incorporates elements of hip-hop. 2. Collected and analyzed data through a cyclical process of observation, analysis, and confirmation.	**RQ1:** Integrate hip-hop music practices into teaching methods, such as sampling, mixing, and improvisation. **RQ2:** Helping students better understand and engage with course content, and developing cross-cultural competencies, better understanding their peers.
3	(30)	USA	Exploring the use of bilingual hip-hop music as a practice in educational contexts.	The research subjects include musicians (aged 28), teachers, and students. No definite age Duration is not clearly stated.	1. Mixed ethnographic approach. 2. (1) In terms of qualitative research, the article used the “mixed ethnography” method combining in-person and digital participant observation. (2) In terms of quantitative research, through an online questionnaire survey.	**RQ1:** Hip-hop music provides them with a channel to express their own voices. **RQ2:** Helping students memorize words and phrases, infer word meanings, practice language fluency, and maintain autonomous interest in learning.
4	(31)	USA	How does hip-hop music hinder or complement students’ learning of statistics?	*n* = 17. High school. 15 white students and 2 Asian students. 2 weeks.	1. Mixed research method. 2. Teachers’ teaching practices and student feedback surveys.	**RQ1:** Using hip-hop music as a teaching tool, integrating it with the content of statistics. **RQ2:** Help elucidate the connection between culture and mathematics, by altering the learning norms and teaching strategies that typically underpin traditional instruction.
5	(17)	USA	Using of hip-hop-based interventions as a teaching and treatment method, applied to STEM (Science, Technology, Engineering, and Mathematics) education, particularly for urban youth of color.	*n* = 10. High school. Primarily Latino and African American. One semester.	1. Qualitative research method. 2. (1) Qualitative research methods, including one-on-one interviews with students, collecting students’ science-themed rap lyrics, and making observational records. (2) The researchers used qualitative coding techniques, such as member checking and thematic coding, to analyze the collected data.	**RQ1:** Writing and performing rap songs with science content, as well as participating in competitions/battles to showcase the written content. **RQ2:**(1) Expressing emotions, gained a significant amount of content knowledge. (2) Reshaping scientific identity and perspectives.
6	(32)	China	Exploring the promoting effect of street dance training on the executive function of preschool children.	*n* = 60. Kindergarten. 4 years old. 30 are in the street dance training group and 30 are in the control group. 8 weeks.	1. Quantitative experimental research method. 2. Pre-test-post-test experimental. Objectively measure and statistically analyze participants’ executive functions.	**RQ1:** Using hip-hop dance as a training component. **RQ2:**8 weeks of street dance training can effectively promote the development of executive function in preschool children.
7	(33)	USA	The effects of hip-hop Instructional Methods on Fifth graders’ Math achievement improvement.	*n* = 113. Fifth grade. Primary school. 52 boys and 61 girls. 4 weeks.	1. Mixed-methods study. 2. Quantitative data (pre-tests-post-tests experimental) and qualitative data (student essays and teacher interviews). Explanatory sequential design, first collecting quantitative data and then collecting qualitative data to further explain the quantitative results.	**RQ1:** Applying hip-hop pedagogy of rap songs, break dance, cypher, and rap battle to the math curriculum. **RQ2:** HHBE can be an effective strategy to improve the math performance of students in high-minority, low-income schools. (1) Rap songs and dance help students memorize key concepts. (2) Sampling strategies allow students to apply existing knowledge to create new knowledge. (3) Cypher and rap battles encourage students to discuss and debate different problem-solving strategies. (4) Math word problems with a hip-hop background increase student learning motivation.
8	(34)	USA	Exploring the challenges and reflections faced by a middle-aged white teacher when teaching a hip-hop culture course.	The author himself. Middle-aged. Duration is not clearly stated.	1. Qualitative research method. 2. Autoethnographic approach. Describing author’s experiences and thought processes in preparing and teaching this hip-hop culture course.	**RQ1:**(1) Asking students to recommend hip-hop songs and analyze the lyrics with them to enhance student engagement and mastery of the course content. (2) Encouraging students to express their personal views more freely, actively reflecting on his own position and perspectives, and openly discussing them in the classroom. **RQ2:** Paying special attention to how their identity and authority are perceived and evaluated by students.
9	(35)	Sweden	Exploring the academization of hip-hop culture and its relationship with teaching.	*n* = 8. 8 famous hip-hop scholars and pioneers. A Swedish hip-hop group “Filthy Dozen Incorporated (FDInc)”. Age 18–28. Duration is not clearly stated.	1. Qualitative research method. 2. The data collection is based on two empirical studies (1) Semi-structured interviews, lasting about 1 hour. 2. (Participant observation and interviews with the Swedish Hip-hop group FDInc, lasting 1 year.	**RQ1:** Using hip-hop culture as a teaching tool. **RQ2:**(1) Hip-hop culture can help solve problems related to race, gender, and class. (2) Providing a platform for students to understand and interpret social realities. (3) Voluntary, open, and improvisational, in contrast to the formal learning formats of schools. (4) The creative processes in hip-hop culture, such as songwriting and dancing, making learning more engaging and enjoyable.
10	(7)	USA	"Why” the use of culturally relevant teaching methods (such as hip-hop) and “how” hip-hop teaching methods affect African American students.	*n* = 2. College. 2 African American students aged 23. 1 boy 1 girl. 1 week.	1. Qualitative research methods, specifically a single case study. 2. Semi-structured interviews and observation methods.	**RQ1:** The professor integrated hip-hop culture and music elements into the course teaching, creating a safe space that allows students to “speak up” and encouraging students to discuss social issues such as racism and gender discrimination. The professor's teaching methods, such as storytelling and incorporating African American vernacular, also help to attract student participation. **RQ2:** (1) Increasing the engagement of African American students. (2) Allowing students to “speak up”.
11	(8)	USA	Exploring the potential and limitations of using hip-hop music as a culturally relevant pedagogy (CRP).	*n* = 20. High school. 13–24. The research does not explicitly mention the specific sample of research subjects, but it involves students in school and youth in the community. Duration is not clearly stated.	1. Qualitative research methods. 2. Data collection through observations and interviews of teacher practices and student responses.	**RQ1:** Hip-hop as a culturally relevant curriculum for urban youth of color, and incorporating hip-hop music into curricula. **RQ2:** (1) Increase student participation and engagement. (2) Foster a community-based cultural atmosphere. (3) Develop students’ critical reading and writing skills. (4) Enhance students’ sense of identity and agency. (5) Create more opportunities for students to express themselves both inside and outside the classroom.
12	(36)	USA	What a male African American kindergarten-first grade teacher learned from the voices of his kindergarten and first-grade students through the use of hip-hop pedagogy and games.	*n* = 13. Private child development center. 5 first-grade students (2 girls, 3 boys) and 8 kindergarten students (5 boys, 3 girls). 11 months.	1. Qualitative research methods, specifically autoethnography. 2. Collecting data includes observations in hip-hop pedagogy/games. Data analysis includes coding, thematic distillation, etc.	**RQ1:** The study uses the culturally relevant method of hip-hop pedagogy, integrating Freestyle rap, hip-hop cultural elements, and hip-hop music into classroom instruction. **RQ2:** It was better able to listen to and understand the voices of the children. This approach helps cultivate children's academic excellence, cultural competence, and critical consciousness.
13	(2)	USA	Exploring whether hip-hop pedagogy can be used as a catalyst for developing leadership and community transformation, both inside and outside the university classroom.	*n* = 25. College. Age between 18-22 years. 10 females and 15 males. One semester.	1. Qualitative research methods, specifically case study. 2. Data collection including participant observation, semi-structured interviews, and course material analysis.	**RQ1:** (1) Create a safe, inclusive, and encouraging creative space for self-expression. (2) Enable students to connect course content with hip-hop music and culture, enhancing the sense of learning significance. (3) Encourages student engagement in the creative process, fostering creativity and civic awareness. **RQ2:** (1) Increasing their engagement and sense of belonging. (2) Helping students find connections between their personal experiences and course content, enhancing the meaningfulness of learning. (3) Cultivates a sense of solidarity and agency through the creative process.
14	(37)	USA	Exploring the benefits of implementing hip-hop pedagogy in an urban science classroom.	*n* = 486. 6th-grade students.68% African American, 26% Latino, 3% Asian, 2% White. Urban middle school. Duration is not clearly stated.	1. Qualitative research methods. 2. Data collection including focus group interviews, video analysis, Likert scale questionnaire, and participant observations and field notes.	**RQ1:** The study including Co-teaching and call-and-response teaching. **RQ2:** (1) Helping students better understand science content and enhance their agency and voice, as these methods are closely related to students’ culture and life. (2) Students expressed that they enjoy listening to hip-hop music in the classroom, as they feel more comfortable and at ease.
15	(38)	USA	The impact of implementing hip-hop pedagogy in urban middle school science classrooms on female students’ teaching and learning.	*n* = 55. Urban middle school. 6th grade. 6 months.	1. Mixed-methods study. (1) Qualitative research methods including student focus group interviews, video clips, and participant observation and field notes. (2) Quantitative research methods including “Increasing students’ interest in STEM” survey questionnaire, analyzing the survey data using Wilcoxon paired *t*-test. 2. Data collection methods include student focus group interviews, video recordings, “Increasing Students’ Interest in STEM” survey questionnaire.	**RQ1:** (1) Hip-hop pedagogy was implemented in the science classroom, such as having students create hip-hop songs to memorize experimental safety rules. (2) Hip-hop-themed interactive activities were designed, such as students acting out molecular simulations of phase changes. **RQ2:** (1) Students gained a deeper understanding of science content. (2) Increased self-confidence. (3) Students developed more interest and positive attitudes towards science courses and teaching methods.

## Findings

### Characteristics of included studies

Thirteen of the included articles (86.7%) originated in the USA while one (6.6%) were from China and one (6.6%) from Sweden.

Nine articles focused on learning experience (60.00%) and three on hip-hop elements in teaching and learning (20.00%). One article concerned hip-hop elements promoting students’ executive functioning (6.6%), including cognitive function, emotional function, and social function. The remainder of the articles (13.33) focused on academization of hip-hop culture and its relationship with teaching (*n* = 1), improve learning by addressing mental health issues (*n* = 1).

Two articles (13.33%) did not specify the age range of participants. This makes research regarding the impact of hip-hop pedagogy on students’ learning effect challenging. The remainder of studies including early childhood to adult, with high school and college students receiving slightly higher weights. This is in line with findings by Broughton (36) who report a similar weighting amongst studies in arts and health.

Hip-hop music as a means of influencing student learning effect in 9 of the 15 articles (60.00%), number of studies utilizing hip-hop culture to influence student learning effect is five (33.33%), and using hip-hop dance as a tool was one (6.66%). Among them, the number of articles using other elements of hip-hop to make an impact on students’ learning effect is two (13.33%). Of the articles focused on hip-hop culture, one (6.66%) did not provide description of the hip-hop pedagogy implementation, while the remaining articles focused on hip-hop based education (*n* = 11), critical pedagogy of hip-hop (*n* = 3).

The literature that met the inclusion criteria utilized a range of article designs, including qualitative (*n* = 9, 60.00%), quantitative (*n* = 2, 13.33%), mixed methods (*n* = 4, 26.67%). Seven of the studies used interviews as a research method (46.67%), three used focus groups interviews (20.00%), and three used questionnaire (20.00%). Nine articles utilized observations (60.00%), two used student feedback (13.33%), two scale test (13.33%), two coding (13.33%), and one autoethnographic (6.66%). Other research methods included video analysis (*n* = 1), field notes (*n* = 1).

Research question 1: What elements of hip-hop pedagogy are appropriate for improving students’ learning effect?

Of the papers included for the investigating how hip-hop pedagogy improves to student's learning effect, 6 articles used hip-hop knowledge, 5 articles used MC, and 4 articles used DJ as a pedagogical component to improve student learning effect. Only 3 articles used cypher, 2 articles used breakdance as pedagogical content, and Graffiti related research was not mentioned. It is worth noting that all 15 articles used hip-hop knowledge as the theoretical basis for the pedagogical content, this phenomenon also echoes Wei et al.'s (6) interpretation of the definition of hip-hop pedagogy:

Hip-hop pedagogy is a learning experience through integrating knowledge related to hip-hop into teaching materials in formal or informal learning environments (p. 05)

**Figure 2:**
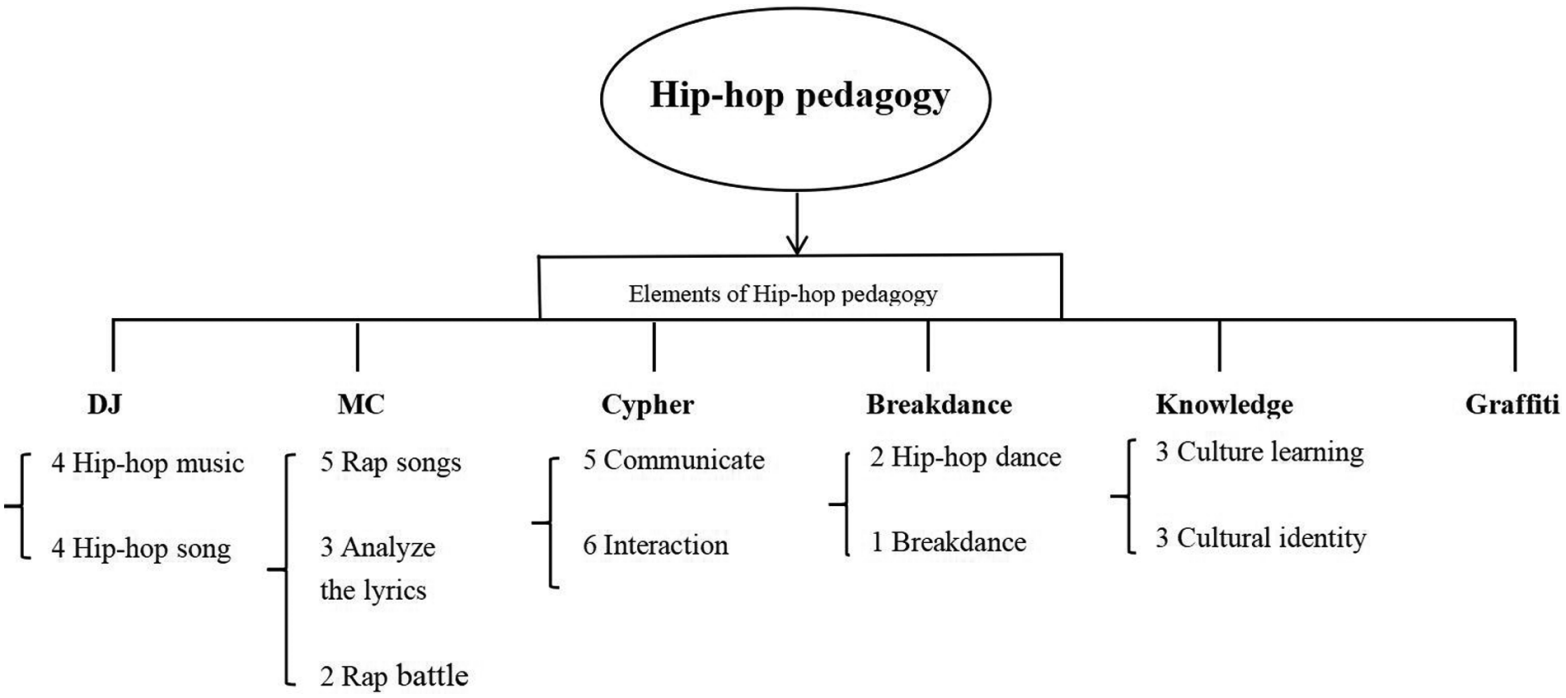
Main review themes for question 1.

This review follows the approach of Chappell et al. (39) on coding main review themes, the articles were analyzed into 6 themes: DJ, MC, cypher, breakdance, knowledge and graffiti (see Figure 2).

#### DJ

Traditionally, a DJ is seen as someone who plays music and mixes (40). In hip-hop pedagogy, the definition of a DJ has expanded, and DJing is more than just hip-hop music, it is a method of teaching (38). Particularly in relation to how hip-hop pedagogy improves to student's learning effect, nine literature confirms the importance of DJ. As one of the well-known hip-hop elements (40), DJ music is widely used in students’ daily learning. Concepts relating to DJ are grouped under hip-hop music and hip-hop song.

A close link between DJ and hip-hop music was identified as improving student learning effect (2, 29). By integrating hip-hop sampling, remixing, and improvisation, students better understand and engage with course content while developing intercultural competence and a better understanding of their peers. Hip-hop Music uses hip-hop music as a teaching tool in conjunction with statistics and math courses (31, 33), providing an outlet for students to express their voices (30).

Several papers correlate using the nature of the curriculum, hip-hop songs are put through compilation or analysis in order to improve students’ learning effect. For example, having students create hip-hop songs to remember lab safety rules (38), or using hip-hop music as a cultural practice that can help students make sense of their world (8), or asking students to recommend hip-hop songs and analyzing the lyrics with them to increase student engagement and mastery of course content (34), or encourage students to create their own “concerts” to deepen their understanding of the content (36).

#### MC

In hip-hop pedagogy, MC is a multifaceted figure who transmits culture, educates students, and fosters community cohesion through rapping and performance (19, 41). MC was identified as a meaningful characteristic of hip-hop pedagogy on students’ learning effect in six peer reviewed, perhaps most in relation to rap songs, analyze the lyrics, and rap battle. In terms of Rap song, different dimensions included bilingual (Aboriginal and English) Rap learning (30), rap songwriting on the theme of scientific content (17), Rap songs to choreograph corresponding hip-hop dance moves, using Rap songs to help students remember and understand the content they have learned, and using hip-hop elements to design mathematical word problems (33), and encouraging students to recite poems they have written on the first day of class to demonstrate their connection to hip-hop culture (34). Some studies also found that “informal classroom materials” were used to reshape the learning environment and that students used Rap to express their interests and knowledge (36).

Przybylski (30) explored by engaging in lyrics analysis, students are better able to memorize the correct words and phrases rather than just mechanically reciting them. Emdin et al. (17) also identified the role played by the use of composed rap lyrics to revise and consolidate knowledge in dealing with exams. Apart from utilizing Rap lyrics to compose knowledge into songs, Greenfield (34) encouraged students to recommend rap songs to be part of the course content, which is a novel attempt to improve learning effect. By having students and teachers act as “MC” and share the content, learning is invariably enhanced during the preparation period (38).

The notion of “MC” related to Rap battle, is a form of debate used to help students explore the best strategies for solving complex math problems. For example, Lutes et al. (33) described the use of Rap Battle to debate math strategies and increase the speed of knowledge integration (17) also noted rap battle as a way to demonstrate learning outcomes and enhance learning through competition.

#### Cypher

Cypher is another focus of hip-hop pedagogy, which Levy et al. (42) describe as “highly codified yet unstructured practices”, for example, “A cipher represents something that is cyclical, such as in freestyle rapping where each participant in the circle takes turns after the other” (p. 104). Given our concern with hip-hop pedagogy, it is not surprising theme of cypher appeared, cypher serves as a non-hip-hop element, but plays the same role of improving students’ learning efficiency. Ten of the fifteen pieces of literature dealt with the introduction of the cypher. Peer reviewed papers foreground the communicate and interaction, sharing is a core element of cypher (33), which coincides with Wei et al.'s (6) view that “Cipher is shared” (p. 04).

Effective communication is built on respect and understanding of students’ cultural backgrounds (35). For example, professor is adept at switching between formal academic vocabulary and everyday slang, using language that students are familiar with to communicate with them (7). Wessel in Wallaert (28) argued that adopting communicate teaching methods, such as role-playing, discussion, and collaborative learning, to meet the learning needs of students, it is also a way of communicating from the student's perspective. Another manifestation of communication is paper exchanges, such as weekly papers containing perceived problems with HHBE implementation (33), students are encouraged to step out of their comfort zones and make connections and understandings with others (2).

Cypher is closely related to the other five elements of hip-hop, and it is a key scene and form for connecting and embodying them. Six of the studies describes the advantages of interact, in addition to gaining knowledge, students gain a deeper understanding of each other and are no longer just strangers in class (2). Not only that, in Kruse (29) study, an open curriculum was introduced where students share their experiences to help other students better understand what they are learning and to improve cross-cultural interacting among students from different backgrounds. Emdin et al. (17) provided a platform for interacting emotions, students are encouraged to discuss sensitive topics such as racism and sexism and to share their views (7). An interesting form of interact comes from Emdin (37) research on a teaching method called “Co-teaching”, which allows students to actively participate in teaching and influencing the content of the classroom. This approach allows students to feel “in control” of the class and to explain and impart knowledge in a more peer-like manner. The same type of interact was seen in the study of Adjapong (38).

#### Breakdance

Of the studies that have utilized elements of dance to improve student's learning effect, we found three pieces of literature that support this idea, including hip-hop dance and breakdance. Hip-hop dance, a more generalized form of popular dance, is often used to interpret hip-hop songs (43). An interesting phenomenon is that the term “Breakdance” has been replaced by the term “Hip-hop dance” in some articles, probably due to the ambiguity of different scholars’ understanding of hip-hop culture (44). Lutes et al. (33) described the use of hip-hop dance in hip-hop pedagogy to enhance student learning, where each rap song is accompanied by dance moves created based on the instructional theme, which helps students to memorize the information in the rap song. Breakdance is the original art expressed in hip-hop culture (45). Adjapong (38) suggested that breakdance improves the understanding and engagement of students, especially female and minority students, in science knowledge through body language communication and hands-on practice. To summarize, There is a difference between “breakdance” and “hip-hop dance”. In the future, breakdance-themed teaching methods could take advantage of dance movements to enhance student learning, for example, students could design a breakdance choreography related to the laws of physics or interpret a story using breakdance movements. In addition, the use of the terms breakdance and hip-hop dance should be defined such that, for example, hip-hop educator the use of breakdance in the study of the five elements of hip-hop and the use of hip-hop within the context of hip-hop culture may be an appropriate choice.

#### Knowledge

In hip-hop education, “knowledge” is defined as the understanding and awareness of hip-hop culture, principles, and social impact, which are shared and preserved through teaching, storytelling, and participation in the community (46, 47). Although all 15 articles refer to “hip-hop knowledge,” “knowledge” in this section is a hip-hop element that should be distinguished from the former. The reason for this phenomenon may be due to the fact that knowledge learning is relatively passive, and may also appear to be more theoretical and abstract, which to affects the number of studies. There are five articles related to knowledge affecting student learning effect in three main aspects: Culture learning, cultural identity. Love (48) argued that knowledge as a youth movement is built on fun, pleasure. Culture learning stimulates students’ goal setting and cultural appreciation, such as focusing on social issues and getting involved on campus (28), and hip-hop culture may be the only area in which students understand and interpret social reality (35). In addition, the interdisciplinary integration of hip-hop culture with other subject areas, such as African oral traditions and the civil rights movement, helps students make connections between knowledge (7).

From a cultural identity perspective, hip-hop pedagogy can be understood as a “culturally relevant pedagogy” (CRP), for example, Kim in Pulido (8) argued that hip-hop music as a cultural practice engages as many youth of color as possible in learning. In music education, teachers who respect hip-hop culture and value its aesthetics will be able to build a sense of cultural identity and communicate more effectively with their students (7). More importantly, students created rap, hip-hop and breakdance based on their understanding of hip-hop knowledge, deepening their learning understanding in the form of “informal classroom materials” (36). Therefore, through the analysis of the above literature, knowledge looks more like a method of instructing students to understand knowledge and acquire it in hip-hop pedagogy.

#### Graffiti

Only one article has explored the role of graffiti in improving student's learning effect. In implementing the hip-hop pedagogy, students are asked to engage in visual art creation, similar to the work of graffiti, as a way of understanding and demonstrating their understanding of science content (38). Although few research has been done on graffiti, the use of graffiti can enhance students’ memorization of formulas and words, as well as promote critical creativity in their work (49, 50). In the future, hip-hop educators will be involved in research on the enhancement of students’ learning effect in the form of image recognition and artistic creation, for example, students’ comprehension and memorization are enhanced by drawing doodles related to the course content. Students are encouraged to reconstruct and represent what they have learned in the form of visual arts to enhance their understanding of complex concepts.

Research question 2: What are the effects of hip-hop pedagogy on students’ learning effect?

**Figure 3:**
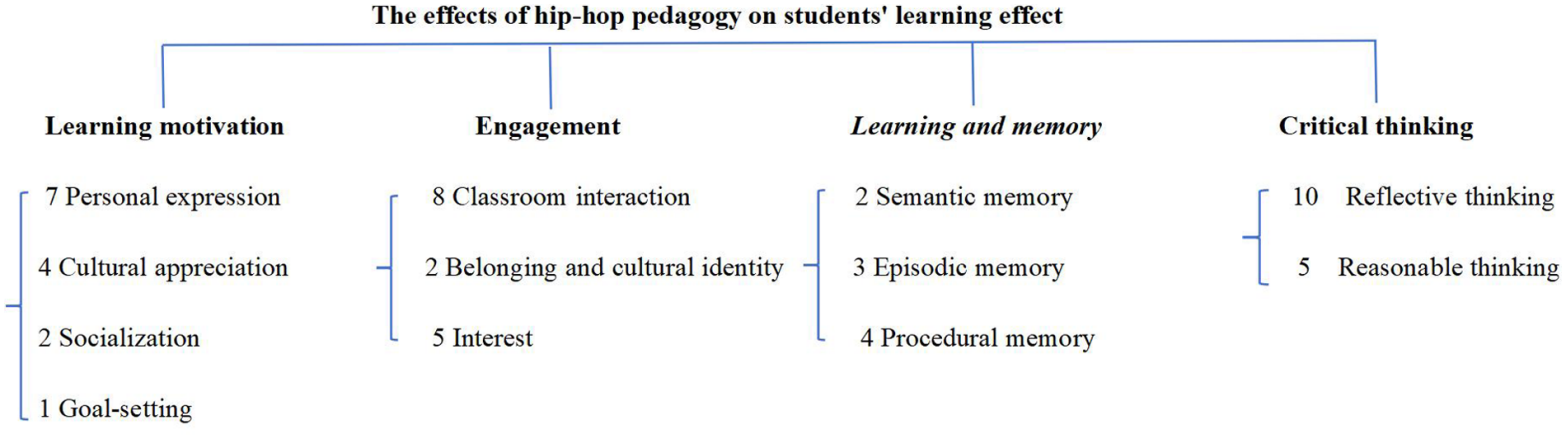
Main review themes for question 2.

Figure 3 illustrates the specific impact of hip-hop pedagogy on improving students’ learning effect. Themes of major impacts (Figure 3) are organized sequentially, and the number of papers in each subtheme is identified so that the reader has a clearer idea of the weighting of each subtheme.

This section describes the positive impact of hip-hop pedagogy on students’ learning effect in four ways: learning motivation, engagement, learning and memory and critical thinking.

#### Learning motivation

Within the thematic of learning motivation, we have grouped concepts which including personal expression, socialization, goal-setting, and cultural appreciation. We found the theme in 11 papers. According to Wessel in Wallaert (28), learning motivation is one of the key manifestations of hip-hop pedagogy to improve student's learning effect.

Regarding personal expression, Hall in Martin's (7) study gives the participant's perspective that “it gives you opportunities to really voice your opinion on what's going on around you” (p. 100). Similar examples are demonstrated in Kruse (29), such as students sharing the song “Not Just a Word” expresses her frustration with her classmates using racially discriminatory language, as well as the process of finally finding the courage to speak up. Two studies report that teacher encouraging students to express themselves personally within the classroom setting significantly boosts their learning motivation (8, 38). When students can share their thoughts, experiences, and creativity, they feel valued and understood, enhancing their intrinsic motivation to learn the course material. Personal expression through activities such as writing, discussions, and creative projects makes learning more relevant and enjoyable, which in turn improves students’ learning effect (2, 7, 17, 33).

Several papers refer to socialization, goal-setting, and cultural appreciation, describing hip-hop pedagogy has taken on the important role of enriching students’ learning experience and thus improving learning effect. Four studies, all with cultural appreciation, discuss improving students’ learning effect, enhancing understanding and participation in course content, developing cross-cultural competencies, fostering engagement and a sense of belonging through creative processes, and connecting personal experiences to the curriculum (29, 35–37). Two studies focused on socialization. Socialization promotes communication among students and enhances learning motivation (2, 28).Some address the foster a community-based cultural atmosphere and increase student autonomy through goal-setting, claiming that this not only boosts motivation but also improves time management and study skills (8).

In summary, socialization, personal expression, goal-setting, and cultural appreciation are key factors that contribute to the learning motivation of students. These elements create a supportive and stimulating learning environment that encourages students to invest more effort and enthusiasm in their studies. As a result, students’ learning effect is significantly improved, leading to better academic outcomes and a more fulfilling educational experience.

#### Engagement

Engagement was identified in 13 of the 15 papers in relation to the classroom interaction, belonging and cultural identity, and interest. The relationship between classroom interaction, improving students’ engagement and learning effect is often considered to be correlated. Several studies have shown that classrooms promote student interaction and improve learning effect through collaboration and group projects (29), helping students understand the connections between culture and mathematics (31). The interactive learning environment encourages students to feel free to discuss and share their ideas (32), for example, hip-hop educator use cypher and rap battles greatly improves students’ learning effect by encouraging them to discuss and debate different problem-solving strategies (33). The use of songwriting and dancing in the classroom changes the dynamics of student-teacher interactions, making them more dynamic and diverse (35). Interactive lectures allow students to actively participate and interact with the material, and this engagement is at the heart of the hip-hop pedagogy (36).In addition, Wessel in Wallaert (28) enhanced student communication and interaction in the classroom through socialization, which is in line with Hall in Martin (7) encouraging multiple forms of expression by creating more opportunities for expression through the instructional methods of student expression.

Factors that also influence student engagement in learning include belonging and cultural identity. According to Kim in Pulido (8), exposed to hip-hop music, students learn how to express their Latino identity and community issues in a way that schools can't provide.“Victoria's counternarrative reaffirms how even in “good” schools, discussions of race and cultural relevance are divorced from academic achievement, the construction of knowledge, and the development of a sociopolitical consciousness” (p. 29). Hip-hop pedagogy aptly makes up for the lack of belonging and cultural identity. Greenfield (34) tried to establish a more equal and interactive classroom atmosphere, allowing students to become co-constructors of knowledge rather than just passive recipients. This approach also helps to promote students’ identity and cultural identity, making them feel that their voices and perspectives are valued and respected in the classroom, it strengthens students’ sense of belonging, solidarity, and meaning.

Five studies demonstrated the use of interest to increase student engagement in learning. For example, using elements of hip-hop pedagogy in the classroom, such as rap battle, rap song, and cypher, helped to capture students’ attention and increase interest and engagement in learning (33, 38). Another example is that the researchers used “performative” assessment, allowing students to participate in hip-hop performances to learn statistical knowledge (31). Interestingly, in some parts of North America, indigenous languages and English are used for bilingual composition of hip-hop music (30). Not only that, but the STEM curriculum has increased students’ interest in learning by encouraging them to demonstrate what they have written by writing and performing rap songs based on science content, as well as participating in competitions/battle (17).

#### Learning and memory

Learning and memory was identified as a meaningful feature of hip-hop pedagogy to improve students’ learning effect, probably mostly related to semantic memory, episodic memory and procedural memory in 8 papers (51). Research related to semantic memory has shown that include memorize words and phrases, infer word meanings, practice language fluency (30), and through Rap song and dance help students to memorize key concepts (33). “It seems clear that the cognitive structure of learning and memory is complex” (52, p. 2).

Several studies have demonstrated a positive relationship between procedural memory and learning and memory. For example, some articles have also found that hip-hop pedagogy prompts students to express emotions, gained a significant amount of content knowledge through repetitive rap songs practice (17, 34), and improving students’ executive functioning through repeatedly practicing hip-hop dance (32).

Several papers identify a positive correlation between hip-hop pedagogy*,* episodic memory *a*nd learning and memory, for example, using hip-hop music as a pedagogical tool in conjunction with a traditional curriculum can help students better understand the content and articulate the cultural and disciplines (29, 31), enriching students’ learning experiences through hip-hop culture to enhance understanding of learning (28), deepening understanding of learning by developing students’ writing skills memorization (8), and teaching through call-and-response (C&R) methods helps students to memorize and understand scientific content (37).

#### Critical thinking

Critical thinking related to all three coded themes (learning motivation, engagement, and learning and memory) was identified as meaningful in 12 of the papers. Critical thinking in hip-hop pedagogy is identified as including reflective and reasonable thinking (53).

Several studies identify a positive correlation between reflective thinking, critical thinking and learning effect. Hip-hop pedagogy helps students remember and understand what they have learned, cultivates students’ reflective thinking, and allows students to use their own background experience to construct new concepts and skills, which helps to cultivate students’ reflective thinking (8, 28, 33–35). Hip-hop culture helping students understand their own world and connect to other social struggles through hip-hop music (7, 17, 29–31).

Several papers found hip-hop pedagogy enhancing and reasonable, including reverse action and changing the formation (32), understand the meaning of hip-hop lyrics from both your own and the writer's standpoint in both directions (17, 34), challenge the existing social order (8), translating hip-hop lyrics in indigenous languages (30), and expressing ideas through rap to address teacher concerns (36).

## Discussion

The purpose of this review was to examine the positive impact of hip-hop pedagogy on students’ learning effect. The subsequent outcomes of the review provide important insights for RQ1 and RQ2, which are analyzed below:

### Discussion regarding research question 1

Overall, the 15 papers offer a rich insight into hip-hop pedagogy improving students’ learning effect clustered around six main forms: DJ, MC, cypher, dance, knowledge and graffiti. We have represented these thematic forms in a tree diagram, but the six thematic forms do not exist in isolation. The analysis shows that there are connections between the boundaries of the thematic forms, such as DJ and dance. In addition, the content of the lower level forms may be a clue to the connections between the higher level forms. For example, hip-hop song and rap song are closely related when lyrics are analyzed, and hip-hop music and Rap battle are simultaneous when applied in practice. This creates a connection between DJ and MC. These interconnections offer us more nuanced insight into the contributions that hip-hop pedagogy makes to students’ learning effect, a certain thematic form may have less existing research, but there are produced effects that are similar to other hip-hop thematic forms, such as graffiti and breakdance (38, 45).

The analysis revealed that the most prevalent thematic forms in hip-hop pedagogy were MC, cypher, and DJ, while fewer studies dealt with breakdance and graffiti. Cypher (mentioned in 10 of 15 articles) is the most classic form of hip-hop pedagogy. Although not included in the five elements of hip-hop, the cypher serves as a vehicle that assumes the functions of the hip-hop elements of interaction and sharing (54), which are core elements of students’ learning effect. DJ (mentioned in 9 out of 15 articles) combines music and learning to enhance student engagement and memorization through musical elements (40). MC (mentioned in 6 out of 15 articles) helped students memorize learning content, encouraged personal expression and fostered critical thinking through rap and lyric analysis (30). There are three reasons why the above three instructional formats have been used in more studies: (1) Ease of use and easy access to equipment. (2) The instructional format is easily integrated with the learning content, e.g., language, formulas. (3) There are no limitations on the level of technology. On the other hand, graffiti and breakdance (55–57), although providing an attractive way for students to learn, may not be able to attract students to participate in general due to the limitation of specialized technology, especially since graffiti materials are not easy to purchase, which may also limit the research.

### Discussion regarding research question 2

This review analyzed four aspects of hip-hop pedagogy's positive impact on students’ learning effect which were ranked as follows out of 15 pieces of literature, according to the percentage of the number of articles:

Learning motivation had the most significant impact effect of all the articles.

This is due to the fact that hip-hop pedagogy enhances students’ desire to learn through a variety of ways, such as personal expression, socialization, goal setting, and cultural appreciation, which in turn enhances students’ learning effect (28, 34). Engagement comes in a close second due to the fact that hip-hop pedagogy emphasizes interaction and participation through group projects, discussions, and performances, making students more active and engaged in the classroom (7, 17). Critical thinking also had a high percentage of effects across all studies, with hip-hop pedagogy enabling students to understand learning content more deeply and analyze it critically through reflection and rational thinking development (8, 29). The relatively low percentage of learning and memory may be due to the fact that hip-hop pedagogy has not yet been fully researched in this area or that its effects are not as significant as in other areas (30, 38), but existing research has demonstrated that learning and memorization promotes students’ learning effect.

## Conclusion and limitation

This review provides readers with descriptions of six themes of hip-hop pedagogy and four positive impacts, in particular, the cypher as a bridge for students to disseminate and share the five elements of hip-hop, demonstrating the invaluable contribution that of hip hop as a form of active living integrated into pedagogy in improving student learning effect is demonstrated.

Although hip-hop pedagogy has shown significant advantages in improving students’ learning efficiency, there are still some shortcomings in this review:

(1) Limit research to a few fixed research themes. Although there have been some studies proving the effect of hip-hop pedagogy, most of them focus on hip-hop music, Rap. more empirical studies on breakdance, graffiti should be carried out in the future in order to validate the long-term effects and the scope of applicability of different forms of hip-hop pedagogy.
(2) Limitations of teaching conditions. Certain forms of hip-hop pedagogy, such as dance and graffiti, are difficult to apply widely in all educational settings due to limitations of venues, resources, and physical abilities. Future research should explore the adaptability and effect of these forms in different educational contexts.

In summary, hip-hop pedagogy has already contributed to the improvement of students’ learning effect through a variety of forms. Although each form has its own unique strengths and challenges, through rational resource allocation, teacher training, and diverse student choices, these forms have the potential for wide application in future education.

## Data Availability

The original contributions presented in the study are included in the article/Supplementary Material, further inquiries can be directed to the corresponding author.
