# Corrigendum: What do we know: positive impact of hip-hop pedagogy on student's learning effects

**DOI:** 10.3389/fspor.2025.1601472

**Published:** 2025-04-15

**Authors:** Xi Ling, Yuanyuan Chen, Shixin Zhao, Dongping Zheng

**Affiliations:** ^1^ Faculty of Education, Silpakorn University, Sanam Chandra Palace Campus, Nakhon Pathom, Thailand; ^2^ Physical Education Department, Fuzhou Institute of Technology, Fuzhou, Fujian, China; ^3^ International Education Center, Thailand National Sports University, Bangkok, Thailand; ^4^ School of Physical Education, Fujian Polytechnic Normal University, Fuzhou, Fujian, China

**Keywords:** positive impact, hip-hop pedagogy, hip-hop culture, student, learning effect

A Corrigendum on: What do we know: positive impact of hip-hop pedagogy on student's learning effects By Ling X, Chen Y, Zhao S, Zheng D (2025). Front. Sports Act. Living 6:1490432. doi: 10.3389/fspor.2024.1490432

**Error in figure/table**

In the published article, there was an error in [FIGURE 1. Literature review and incorporation flowchart.] as published. [The contents of the figure from the previous edition]. The corrected (Figure 1) and its caption **[Literature review and incorporation flowchart] appear below.

The authors apologize for this error and state that this does not change the scientific conclusions of the article in any way. The original article has been updated.

**Text correction**

**Figure 1:**
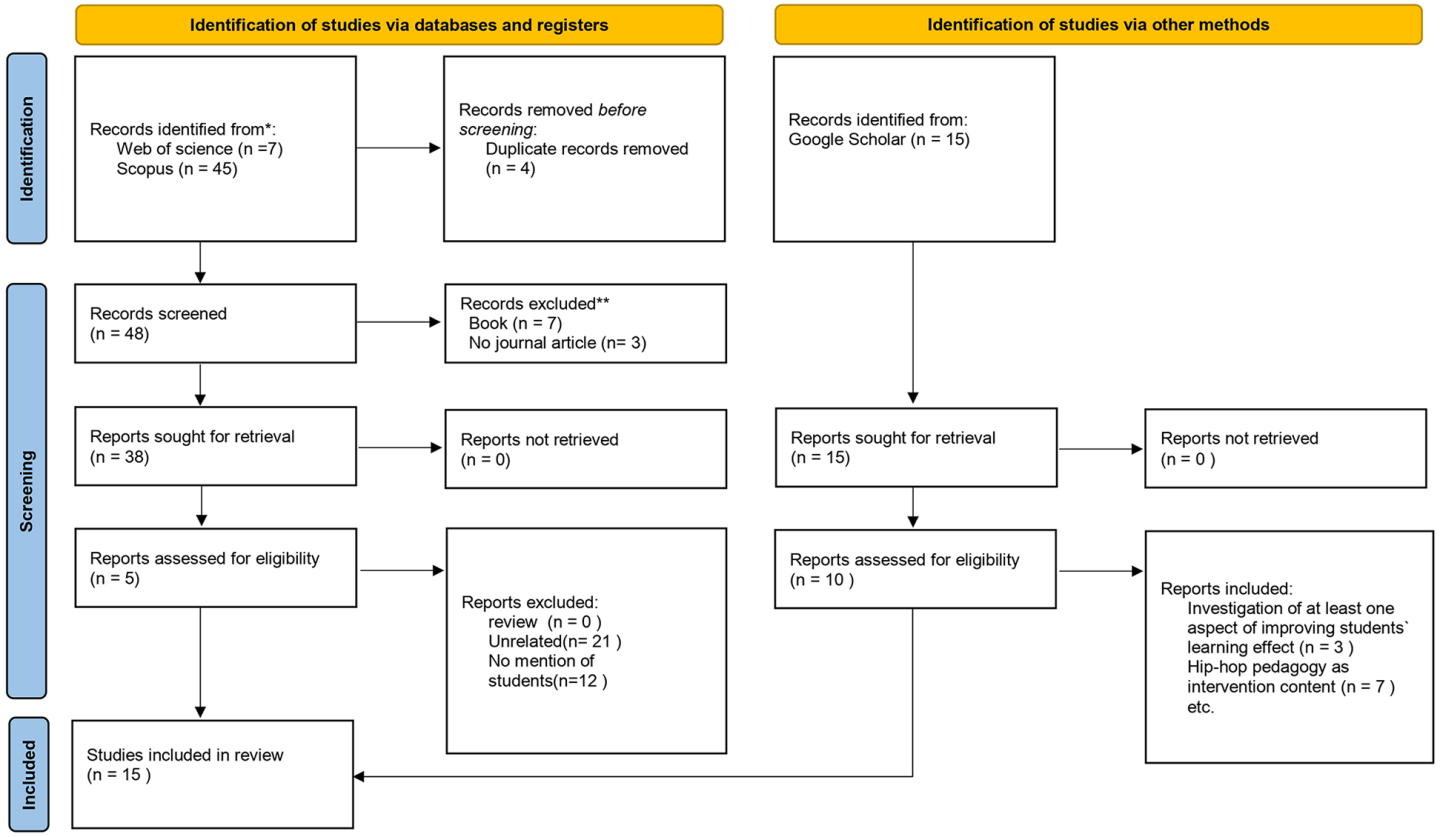
Literature review and incorporation flowchart.

In the published article, there was an error. [Incorrectly entered numbers].

A correction has been made to [Result], [Paragraph 9]. This sentence previously stated:

“and after screening for inclusion criteria, 7 articles were selected.”

The corrected sentence appears below:

“and after screening for inclusion criteria, 10 articles were selected.”

The authors apologize for this error and state that this does not change the scientific conclusions of the article in any way. The original article has been updated.

